# 2-[4-(Morpholin-4-ylmeth­yl)phen­yl]benzonitrile

**DOI:** 10.1107/S1600536812050957

**Published:** 2012-12-22

**Authors:** Gangadhar Y. Meti, R. R Kamble, A. J. Ravi, H. K. Arunkashi, H. C. Devarajegowda

**Affiliations:** a Department of Studies in Chemistry, Karnataka University, Dharwad 580 003, Karnataka, India; b Department of Physics, Yuvaraja’s College (Constituent College), University of Mysore, Mysore 570 005, Karnataka, India

## Abstract

In the title compound, C_18_H_18_N_2_O, the morpholine ring adopts a chair conformation with the exocyclic N—C bond in an equatorial orientation. The dihedral angles between the central benzene ring and the morpholine ring (all atoms) and the cyano­benzene ring are 87.87 (7) and 52.54 (7)°, respectively. No significant inter­molecular inter­actions are observed in the crystal structure.

## Related literature

For biological applications of biphenyl derivatives see; Li *et al.* (2011 ▶); Hadizad *et al.* (2009 ▶); Larsen *et al.* (1994 ▶); Kamble *et al.* (2011 ▶); Zhang *et al.* (2004 ▶); Chan *et al.* (1994 ▶).
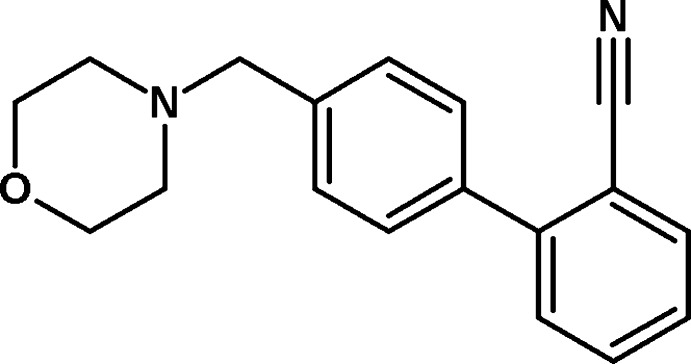


## Experimental

### 

#### Crystal data

C_18_H_18_N_2_O
*M* *_r_* = 278.34
Monoclinic, 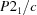

*a* = 21.1079 (5) Å
*b* = 8.1358 (1) Å
*c* = 9.0793 (2) Å
β = 100.833 (1)°
*V* = 1531.40 (5) Å^3^
*Z* = 4
Mo *K*α radiation
μ = 0.08 mm^−1^
*T* = 296 K
0.24 × 0.20 × 0.12 mm

#### Data collection

Bruker SMART CCD diffractometer
Absorption correction: multi-scan (*SADABS*; Sheldrick, 2007 ▶) *T*_min_ = 0.770, *T*_max_ = 1.000
10319 measured reflections
2387 independent reflections
1948 reflections with *I* > 2σ(*I*)
*R*_int_ = 0.021

#### Refinement

*R*[*F* ^2^ > 2σ(*F* ^2^)] = 0.033
*wR*(*F* ^2^) = 0.083
*S* = 1.03
2387 reflections
191 parameters
H-atom parameters constrained
Δρ_max_ = 0.11 e Å^−3^
Δρ_min_ = −0.10 e Å^−3^

### 

Data collection: *SMART* (Bruker, 2001 ▶); cell refinement: *SAINT* (Bruker, 2001 ▶); data reduction: *SAINT*; program(s) used to solve structure: *SHELXS97* (Sheldrick, 2008 ▶); program(s) used to refine structure: *SHELXL97* (Sheldrick, 2008 ▶); molecular graphics: *ORTEP-3* (Farrugia, 2012 ▶); software used to prepare material for publication: *SHELXL97*.

## Supplementary Material

Crystal structure: contains datablock(s) I, global. DOI: 10.1107/S1600536812050957/hb7014sup1.cif

Structure factors: contains datablock(s) I. DOI: 10.1107/S1600536812050957/hb7014Isup2.hkl

Supplementary material file. DOI: 10.1107/S1600536812050957/hb7014Isup3.cml

Additional supplementary materials:  crystallographic information; 3D view; checkCIF report

## Figures and Tables

**Fig. 1.**
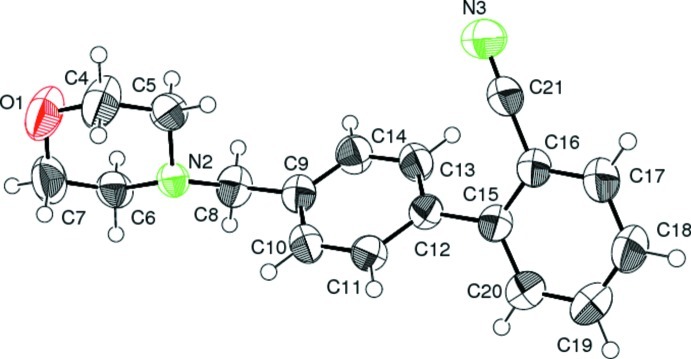
The molecular structure of the title compound. Displacement ellipsoids are drawn at the 50% probability level. Hydrogen atoms are shown as spheres of arbitrary radius.

**Fig. 2.**
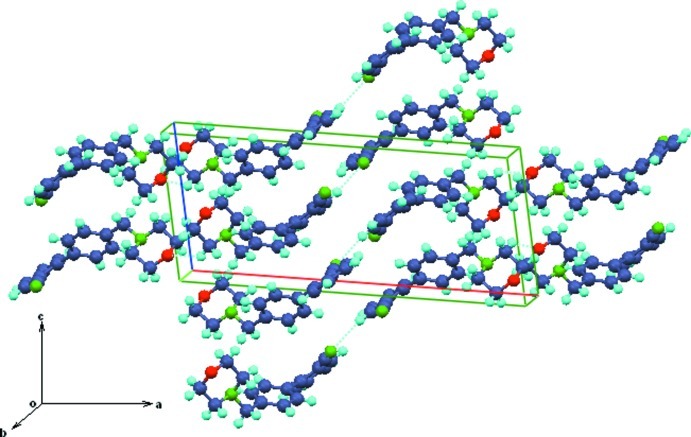
The packing of molecules.

## supplementary crystallographic information

### Comment

Biphenyl derivatives were reported as non-peptide A^II^ antagonists by the discovery at Du-pont Merck which resulted into clinical candidate (5–2-[(4-methyl)-biphenyl]-1*H*-tetrazole. This has become the common motif for most of the potent antagonists reported (Li *et al.*, 2011; Hadizad *et al.*, 2009; Larsen *et al.*, 1994). This discovery lead to the development of drugs such as irbesartan and losartan for the efficient treatment of hypertension. Since then these biphenyl derivatives have received enormous focus due to their inhibition of angiotensin converting enzyme (ACE) and in this regard many biphenyl derivatives have been reported (Kamble *et al.*, 2011; Zhang *et al.*, 2004; Chan *et al.*, 1994).

The asymmetric unit of 4'-(morpholin-4-ylmethyl)biphenyl-2-carbonitrile is shown in Fig. 1. The the morpholine ring (O1/N2/C4–C7)adopts a chair conformation. The dihedral angle between the morpholine ring (O1/N2/C4–C7) and the benzene rings (C9–C14) and (C15–C20) are 87.87 (7)° and 44.76 (7)° respectively. No significant intermolecular interactions are observed. The crystal packing of the molecules is shown in Fig.2.

### Experimental

A mixture of 4'-(bromomethyl)-biphenyl-2-carbonitrile (0.0074 mol), morpholine (0.0085 mol) in presence of potassium carbonate (0.009 mol) in acetone (20 ml) was stirred at 298–300 K for 5–6hrs, filtred the salt, filtrate added to 50 ml water stirred well to get solid, filtered and washed with water, dried at 313 K. Colourless plates were recrystallized from a solvent mixture of acetone and THF (m.p. 348 K).

### Refinement

All H atoms were positioned geometrically, with C—H = 0.93 Å for aromatic H and C—H = 0.97 Å for methylene H and refined using a riding model with *U*_iso_(H) = 1.2*U*_eq_(C) for aromatic and methylene H.

### Crystal data

**Table:**

C_18_H_18_N_2_O	*F*(000) = 592
*M**_r_* = 278.34	*D*_x_ = 1.207 Mg m^−^^3^
Monoclinic, *P*2_1_/*c*	Melting point: 348 K
Hall symbol: -P 2ybc	Mo *K*α radiation, λ = 0.71073 Å
*a* = 21.1079 (5) Å	Cell parameters from 2387 reflections
*b* = 8.1358 (1) Å	θ = 2.7–24.1°
*c* = 9.0793 (2) Å	µ = 0.08 mm^−^^1^
β = 100.833 (1)°	*T* = 296 K
*V* = 1531.40 (5) Å^3^	Plate, colourless
*Z* = 4	0.24 × 0.20 × 0.12 mm

### Data collection

**Table:**

Bruker SMART CCD diffractometer	2387 independent reflections
Radiation source: fine-focus sealed tube	1948 reflections with *I* > 2σ(*I*)
Graphite monochromator	*R*_int_ = 0.021
ω and φ scans	θ_max_ = 24.1°, θ_min_ = 2.7°
Absorption correction: multi-scan (*SADABS*; Sheldrick, 2007)	*h* = −18→24
*T*_min_ = 0.770, *T*_max_ = 1.000	*k* = −9→9
10319 measured reflections	*l* = −10→10

### Refinement

**Table:**

Refinement on *F*^2^	Secondary atom site location: difference Fourier map
Least-squares matrix: full	Hydrogen site location: inferred from neighbouring sites
*R*[*F*^2^ > 2σ(*F*^2^)] = 0.033	H-atom parameters constrained
*wR*(*F*^2^) = 0.083	*w* = 1/[σ^2^(*F*_o_^2^) + (0.0398*P*)^2^ + 0.1412*P*] where *P* = (*F*_o_^2^ + 2*F*_c_^2^)/3
*S* = 1.03	(Δ/σ)_max_ < 0.001
2387 reflections	Δρ_max_ = 0.11 e Å^−^^3^
191 parameters	Δρ_min_ = −0.10 e Å^−^^3^
0 restraints	Extinction correction: *SHELXL97* (Sheldrick, 2008), Fc^*^=kFc[1+0.001xFc^2^λ^3^/sin(2θ)]^-1/4^
Primary atom site location: structure-invariant direct methods	Extinction coefficient: 0.0110 (13)

### Special details

**Table:**

Experimental. Spectroscopic data IR (KBr): 3040–3080, 2175, 1500, ^1^H NMR (300 MHz, CDCl_3_, δ p.p.m..): 2.93 (t,4*H*, Morpholine CH_2_), 3.8 (s, 2H, CH_2_), 3.94 (t, 4H, morpholine CH_2_), 7.42- 7.84 (m, 8H, ArH). MS (m/*z*, 70 eV): 278, 250, 192.
Geometry. All s.u.'s (except the s.u. in the dihedral angle between two l.s. planes) are estimated using the full covariance matrix. The cell s.u.'s are taken into account individually in the estimation of s.u.'s in distances, angles and torsion angles; correlations between s.u.'s in cell parameters are only used when they are defined by crystal symmetry. An approximate (isotropic) treatment of cell s.u.'s is used for estimating s.u.'s involving l.s. planes.
Refinement. Refinement of *F*^2^ against ALL reflections. The weighted *R*-factor *wR* and goodness of fit *S* are based on *F*^2^, conventional *R*-factors *R* are based on *F*, with *F* set to zero for negative *F*^2^. The threshold expression of *F*^2^ > 2σ(*F*^2^) is used only for calculating *R*-factors(gt) *etc*. and is not relevant to the choice of reflections for refinement. *R*-factors based on *F*^2^ are statistically about twice as large as those based on *F*, and *R*- factors based on ALL data will be even larger.

### Fractional atomic coordinates and isotropic or equivalent isotropic displacement parameters (Å^2^)

**Table:**

	*x*	*y*	*z*	*U*_iso_*/*U*_eq_	
O1	0.05263 (6)	0.66354 (15)	0.88307 (13)	0.0926 (4)	
N2	0.11430 (4)	0.39688 (12)	0.76422 (10)	0.0462 (3)	
N3	0.42812 (7)	0.18166 (17)	1.11979 (17)	0.0859 (4)	
C4	0.10594 (8)	0.5807 (2)	0.96979 (18)	0.0835 (5)	
H4A	0.0904	0.4927	1.0258	0.100*	
H4B	0.1302	0.6568	1.0412	0.100*	
C5	0.14930 (6)	0.51072 (17)	0.87289 (15)	0.0584 (4)	
H5A	0.1667	0.5991	0.8209	0.070*	
H5B	0.1851	0.4541	0.9351	0.070*	
C6	0.05896 (6)	0.48231 (17)	0.67698 (15)	0.0585 (4)	
H6A	0.0337	0.4060	0.6075	0.070*	
H6B	0.0739	0.5695	0.6191	0.070*	
C7	0.01763 (7)	0.5536 (2)	0.7778 (2)	0.0789 (5)	
H7A	−0.0185	0.6112	0.7178	0.095*	
H7B	0.0005	0.4654	0.8306	0.095*	
C8	0.15600 (7)	0.33485 (18)	0.66566 (14)	0.0583 (4)	
H8A	0.1782	0.4270	0.6304	0.070*	
H8B	0.1293	0.2843	0.5788	0.070*	
C9	0.20548 (6)	0.21152 (16)	0.73752 (13)	0.0504 (3)	
C10	0.18769 (6)	0.07434 (16)	0.81104 (13)	0.0523 (3)	
H10	0.1448	0.0608	0.8197	0.063*	
C11	0.23247 (6)	−0.04191 (16)	0.87128 (13)	0.0507 (3)	
H11	0.2193	−0.1331	0.9194	0.061*	
C12	0.29698 (6)	−0.02548 (15)	0.86153 (14)	0.0491 (3)	
C13	0.31476 (6)	0.11113 (16)	0.78798 (15)	0.0582 (4)	
H13	0.3577	0.1251	0.7798	0.070*	
C14	0.26966 (6)	0.22674 (17)	0.72675 (15)	0.0585 (4)	
H14	0.2827	0.3170	0.6771	0.070*	
C15	0.34412 (6)	−0.15362 (15)	0.92802 (15)	0.0527 (3)	
C16	0.40058 (6)	−0.11543 (16)	1.02980 (16)	0.0566 (4)	
C17	0.44281 (7)	−0.23758 (18)	1.09399 (18)	0.0706 (4)	
H17	0.4801	−0.2099	1.1615	0.085*	
C18	0.42949 (8)	−0.39895 (19)	1.0579 (2)	0.0776 (5)	
H18	0.4578	−0.4810	1.1006	0.093*	
C19	0.37458 (8)	−0.43904 (18)	0.9591 (2)	0.0789 (5)	
H19	0.3655	−0.5487	0.9351	0.095*	
C20	0.33248 (7)	−0.31866 (17)	0.89470 (18)	0.0687 (4)	
H20	0.2954	−0.3486	0.8274	0.082*	
C21	0.41517 (6)	0.05194 (18)	1.07696 (17)	0.0633 (4)	

### Atomic displacement parameters (Å^2^)

**Table:**

	*U*^11^	*U*^22^	*U*^33^	*U*^12^	*U*^13^	*U*^23^
O1	0.0948 (8)	0.0848 (8)	0.0920 (8)	0.0435 (7)	0.0019 (7)	−0.0129 (6)
N2	0.0412 (6)	0.0469 (6)	0.0480 (6)	0.0037 (4)	0.0018 (4)	0.0029 (4)
N3	0.0726 (9)	0.0598 (9)	0.1148 (11)	−0.0027 (7)	−0.0098 (8)	−0.0077 (8)
C4	0.0912 (12)	0.0866 (11)	0.0668 (9)	0.0383 (9)	−0.0004 (9)	−0.0144 (9)
C5	0.0537 (8)	0.0582 (8)	0.0572 (8)	0.0045 (6)	−0.0051 (6)	−0.0016 (6)
C6	0.0477 (7)	0.0567 (8)	0.0651 (8)	0.0028 (6)	−0.0051 (6)	0.0076 (7)
C7	0.0517 (9)	0.0840 (12)	0.0987 (12)	0.0207 (8)	0.0079 (8)	0.0104 (10)
C8	0.0629 (8)	0.0654 (9)	0.0447 (7)	0.0125 (7)	0.0054 (6)	0.0041 (6)
C9	0.0529 (8)	0.0578 (8)	0.0394 (6)	0.0078 (6)	0.0062 (5)	−0.0035 (6)
C10	0.0434 (7)	0.0646 (8)	0.0488 (7)	0.0043 (6)	0.0081 (6)	−0.0036 (6)
C11	0.0491 (7)	0.0507 (7)	0.0525 (7)	−0.0014 (6)	0.0101 (6)	−0.0021 (6)
C12	0.0475 (7)	0.0463 (7)	0.0529 (7)	0.0029 (6)	0.0079 (6)	−0.0050 (6)
C13	0.0453 (7)	0.0593 (8)	0.0710 (8)	0.0027 (6)	0.0137 (6)	0.0035 (7)
C14	0.0578 (9)	0.0567 (8)	0.0618 (8)	0.0030 (7)	0.0132 (7)	0.0087 (7)
C15	0.0488 (7)	0.0467 (7)	0.0638 (8)	0.0037 (6)	0.0139 (6)	−0.0009 (6)
C16	0.0466 (8)	0.0482 (8)	0.0746 (9)	0.0051 (6)	0.0106 (7)	0.0021 (7)
C17	0.0530 (8)	0.0618 (9)	0.0932 (11)	0.0106 (7)	0.0044 (8)	0.0069 (8)
C18	0.0693 (10)	0.0554 (9)	0.1073 (12)	0.0191 (8)	0.0140 (9)	0.0113 (9)
C19	0.0840 (11)	0.0446 (8)	0.1079 (12)	0.0080 (8)	0.0171 (10)	−0.0030 (8)
C20	0.0637 (9)	0.0522 (9)	0.0874 (10)	0.0010 (7)	0.0070 (8)	−0.0079 (8)
C21	0.0458 (8)	0.0573 (9)	0.0821 (10)	0.0039 (7)	−0.0003 (7)	0.0027 (8)

### Geometric parameters (Å, º)

**Table:**

O1—C7	1.4120 (19)	C10—C11	1.3758 (17)
O1—C4	1.4169 (18)	C10—H10	0.9300
N2—C5	1.4502 (16)	C11—C12	1.3873 (17)
N2—C8	1.4581 (16)	C11—H11	0.9300
N2—C6	1.4585 (15)	C12—C13	1.3843 (18)
N3—C21	1.1401 (18)	C12—C15	1.4886 (18)
C4—C5	1.496 (2)	C13—C14	1.3788 (18)
C4—H4A	0.9700	C13—H13	0.9300
C4—H4B	0.9700	C14—H14	0.9300
C5—H5A	0.9700	C15—C20	1.3881 (19)
C5—H5B	0.9700	C15—C16	1.3989 (18)
C6—C7	1.495 (2)	C16—C17	1.3880 (18)
C6—H6A	0.9700	C16—C21	1.443 (2)
C6—H6B	0.9700	C17—C18	1.369 (2)
C7—H7A	0.9700	C17—H17	0.9300
C7—H7B	0.9700	C18—C19	1.365 (2)
C8—C9	1.5057 (17)	C18—H18	0.9300
C8—H8A	0.9700	C19—C20	1.377 (2)
C8—H8B	0.9700	C19—H19	0.9300
C9—C14	1.3819 (18)	C20—H20	0.9300
C9—C10	1.3879 (18)		
			
C7—O1—C4	109.55 (11)	C10—C9—C8	121.06 (12)
C5—N2—C8	110.46 (10)	C11—C10—C9	121.07 (12)
C5—N2—C6	108.59 (10)	C11—C10—H10	119.5
C8—N2—C6	110.24 (9)	C9—C10—H10	119.5
O1—C4—C5	111.35 (12)	C10—C11—C12	121.17 (12)
O1—C4—H4A	109.4	C10—C11—H11	119.4
C5—C4—H4A	109.4	C12—C11—H11	119.4
O1—C4—H4B	109.4	C13—C12—C11	117.81 (12)
C5—C4—H4B	109.4	C13—C12—C15	122.46 (11)
H4A—C4—H4B	108.0	C11—C12—C15	119.73 (11)
N2—C5—C4	110.69 (12)	C14—C13—C12	120.86 (12)
N2—C5—H5A	109.5	C14—C13—H13	119.6
C4—C5—H5A	109.5	C12—C13—H13	119.6
N2—C5—H5B	109.5	C13—C14—C9	121.47 (13)
C4—C5—H5B	109.5	C13—C14—H14	119.3
H5A—C5—H5B	108.1	C9—C14—H14	119.3
N2—C6—C7	110.63 (11)	C20—C15—C16	116.92 (12)
N2—C6—H6A	109.5	C20—C15—C12	120.77 (12)
C7—C6—H6A	109.5	C16—C15—C12	122.27 (11)
N2—C6—H6B	109.5	C17—C16—C15	121.23 (13)
C7—C6—H6B	109.5	C17—C16—C21	117.81 (13)
H6A—C6—H6B	108.1	C15—C16—C21	120.89 (11)
O1—C7—C6	111.65 (12)	C18—C17—C16	119.94 (15)
O1—C7—H7A	109.3	C18—C17—H17	120.0
C6—C7—H7A	109.3	C16—C17—H17	120.0
O1—C7—H7B	109.3	C19—C18—C17	119.81 (14)
C6—C7—H7B	109.3	C19—C18—H18	120.1
H7A—C7—H7B	108.0	C17—C18—H18	120.1
N2—C8—C9	114.47 (10)	C18—C19—C20	120.64 (14)
N2—C8—H8A	108.6	C18—C19—H19	119.7
C9—C8—H8A	108.6	C20—C19—H19	119.7
N2—C8—H8B	108.6	C19—C20—C15	121.46 (14)
C9—C8—H8B	108.6	C19—C20—H20	119.3
H8A—C8—H8B	107.6	C15—C20—H20	119.3
C14—C9—C10	117.62 (12)	N3—C21—C16	177.11 (16)
C14—C9—C8	121.26 (12)		
			
C7—O1—C4—C5	58.34 (19)	C10—C9—C14—C13	−0.67 (19)
C8—N2—C5—C4	177.40 (11)	C8—C9—C14—C13	−177.75 (12)
C6—N2—C5—C4	56.40 (14)	C13—C12—C15—C20	−128.35 (15)
O1—C4—C5—N2	−58.71 (18)	C11—C12—C15—C20	51.08 (18)
C5—N2—C6—C7	−56.05 (15)	C13—C12—C15—C16	53.85 (18)
C8—N2—C6—C7	−177.18 (12)	C11—C12—C15—C16	−126.71 (14)
C4—O1—C7—C6	−58.19 (17)	C20—C15—C16—C17	0.0 (2)
N2—C6—C7—O1	58.11 (16)	C12—C15—C16—C17	177.92 (13)
C5—N2—C8—C9	73.85 (14)	C20—C15—C16—C21	−176.91 (13)
C6—N2—C8—C9	−166.14 (11)	C12—C15—C16—C21	1.0 (2)
N2—C8—C9—C14	−130.98 (13)	C15—C16—C17—C18	0.0 (2)
N2—C8—C9—C10	52.04 (16)	C21—C16—C17—C18	177.05 (15)
C14—C9—C10—C11	0.17 (18)	C16—C17—C18—C19	−0.2 (3)
C8—C9—C10—C11	177.25 (11)	C17—C18—C19—C20	0.3 (3)
C9—C10—C11—C12	0.45 (18)	C18—C19—C20—C15	−0.2 (2)
C10—C11—C12—C13	−0.56 (18)	C16—C15—C20—C19	0.1 (2)
C10—C11—C12—C15	179.98 (11)	C12—C15—C20—C19	−177.84 (13)
C11—C12—C13—C14	0.05 (19)	C17—C16—C21—N3	−25 (3)
C15—C12—C13—C14	179.50 (12)	C15—C16—C21—N3	152 (3)
C12—C13—C14—C9	0.6 (2)		
